# Tolerance of a Vascularized Composite Allograft Achieved in MHC Class-I-mismatch Swine *via* Mixed Chimerism

**DOI:** 10.3389/fimmu.2022.829406

**Published:** 2022-05-10

**Authors:** Alexandre G. Lellouch, Alec R. Andrews, Gaelle Saviane, Zhi Yang Ng, Ilse M. Schol, Marion Goutard, Amon-Ra Gama, Ivy A. Rosales, Robert B. Colvin, Laurent A. Lantieri, Mark A. Randolph, Gilles Benichou, Curtis L. Cetrulo

**Affiliations:** ^1^ Division of Plastic and Reconstructive Surgery, Massachusetts General Hospital, Harvard Medical School, Boston, MA, United States; ^2^ Vascularized Composite Allotransplantation Laboratory, Center for Transplantation Sciences, Massachusetts General Hospital, Harvard Medical School, Boston, MA, United States; ^3^ Service de Chirurgie Plastique, Hôpital Européen Georges Pompidou, Assistance Publique-Hôpitaux de Paris (APHP), Université Paris Descartes, Paris, France; ^4^ Shriners Hospitals for Children, Harvard Medical School, Boston, MA, United States; ^5^ Center for Transplantation Sciences, Department of Surgery, Massachusetts General Hospital, Harvard Medical School, Boston, MA, United States

**Keywords:** Vascularized composite allotransplantation (VCA), bone marrow transplantation, mixed chimerism, co-stimulatory blockade, skin tolerance, MHC class I

## Abstract

**Background:**

Vascularized composite allografts (VCAs) allow reconstruction of devastating injuries and amputations, yet require lifelong immunosuppression that is associated with significant morbidity. Induction of immune tolerance of VCAs would permit widespread use of these procedures. VCAs are acquired from deceased donors most likely to be *fully*-MHC-mismatched (in contrast to living-related renal transplant donor-recipient pairs matched at one MHC haplotype). After achieving VCA tolerance in a swine model equivalent to clinical living-related renal transplants (single-haplotype MHC mismatches: e.g., “mother-daughter”/haploidentical), we tested our protocol in MHC class I, class II, and fully-MHC-mismatched pairs. Although class II mismatched swine demonstrated similar results as the haploidentical scenario (stable mixed chimerism and tolerance), our protocol failed to prevent rejection of class I and full mismatch VCAs. Here, we describe a new adapted conditioning protocol that successfully achieved tolerance across MHC class-I-mismatch barriers in swine.

**Methods:**

Swine were treated with non-myeloablative total body and thymic irradiation two days prior to infusion of bone marrow cells from an MHC class I-mismatched donor. They also received a short-term treatment with CTLA4-Ig (Belatacept^®^) and anti-IL6R mAb (Tociluzimab^®^) and were transplanted with an osteomyocutaneous VCA from the same donor.

**Results:**

Stable mixed chimerism and tolerance of MHC class-I-mismatched VCAs was achieved in 3 recipients. Allograft tolerance was associated with a sustained lack of anti-donor T cell response and a concomitant expansion of double negative CD4^-^CD8^-^ T cells producing IL-10.

**Conclusions:**

This study demonstrates the first successful mixed chimerism-induced VCA tolerance in a large animal model across a MHC class-I-mismatch. Future studies aimed at fully-mismatched donor-recipient pairs are under investigation with this protocol.

## Introduction

Since the first successful hand transplant was performed in France in 1998 (1) the field of vascularized composite allotransplantation (VCA) has progressed to a collective, worldwide experience of more than 120 hand transplants, 46 face transplants (2) and a myriad of other VCAs including lower limb, abdominal wall, penis, laryngeal, scalp and even uterine transplants (3). These remarkable procedures have revolutionized reconstructive surgery with unprecedented outcomes in aesthetic form and functional return after devastating injury or cancer ablation. As with all allotransplants including life-saving solid organ transplants, however, long-term systemic immunosuppression is required to prevent allograft rejection, imparting a risk of significant morbidity that includes metabolic, infectious, renovascular and neoplastic complications (4, 5).

In order to offer widespread application of these procedures, we and others have pursued the induction of immunologic tolerance by establishing mixed hematopoietic chimerism (6, 7); a state in which the transplant recipient’s hematopoietic system consists of both donor and recipient elements, such that the transplanted allograft is recognized as “self” and thereby permanently accepted after the cessation of immunosuppression. Mixed chimerism protocols developed in the genetically defined Massachusetts General Hospital (MGH) swine model for *renal* transplantation were successfully translated to the clinic for living-related donor-recipient kidney transplant pairs with single haplotype major histocompatibility complex (MHC) mismatches (haploidentical) (8). Following this blueprint, our laboratory developed a similar protocol for VCA in MGH swine obtaining stable mixed chimerism and long-term VCA tolerance through VCA and co-infusion of cytokine-mobilized peripheral blood donor stem cells (CM-PBMCs) from a haploidentical donor (9).

Clinically, VCAs are acquired from *deceased* donors that are most likely to be *fully* MHC mismatched (unlike a living-related renal transplant donor-recipient pair that are matched at one MHC haplotype). After achieving success in a preclinical VCA swine model equivalent to clinical living-related renal transplant results (single-haplotype MHC mismatches: e.g., “mother-daughter”/haploidentical), we proceeded to test this protocol on fully MHC-mismatched swine pairs. However, we observed either poor stem cell engraftment and only transient chimerism that was insufficient for tolerance, or morbidity secondary to the requirement for harsh myeloablative conditioning.

Using controlled mismatches available with the MGH swine herd, we attempted to tease out the swine leukocyte antigens (SLA) responsible for the failure in fully mismatched pairs and tested the previously successful haploidentical protocol using CM-PBMCs in both MHC class I-matched/class II-mismatched barriers and vice versa (i.e. MHC class I-mismatched/class II-matched barriers). In these studies, the class II mismatched results mirrored the haploidentical transplant results; the CM-PBMC stem cell source resulted in stable mixed chimerism and tolerance. Surprisingly, even though we obtained stable mixed chimerism in the class I-mismatches, the VCAs were rejected (10).

To solve the class-I-mismatch rejection problem, we made the following changes to our regimen: we moderately increased irradiation of the whole body and thymus, replaced cyclosporine with FK506, added co-stimulatory blockade with CTLA4-Ig, added anti-IL6R mAb and added a vascularized bone marrow component to our VCA by including the femur in the transplanted tissue. With this regimen, we achieved reproducible, stable mixed chimerism for the first-time across a class I mismatch barrier in a large animal model, demonstrating that tolerance to VCA is possible in a clinically relevant large animal model. Importantly, we achieved stable mixed chimerism with use of donor bone marrow as our stem cell source (rather than CM-PBMCs) which resulted in long-term immunosuppression-free tolerance of all components of the VCAs including the epidermis. Additionally, all conditioning medications (FK506, CTLA4-Ig, steroids, and anti-IL6R mAb) employed in this study are FDA-approved for clinical use in various other conditions, and while the protocol tested here was not intended for clinical readiness, these attributes (donor bone marrow and approved drugs), taken together, will be valuable for eventual clinical tolerance protocols across full deceased donor-recipient mismatches (mismatched at class I and class II loci).

## Materials and Methods

### Animals

All animal care and procedures were approved by the MGH Institutional Animal Care and Use Committee (IACUC) and conducted in compliance with the *Guide for the Care and Use of Laboratory Animals* prepared by the Institute of Laboratory Animal Resources, National Research Council, and published by the National Academy Press. This study utilized the MGH miniature swine model (11). Lines of MGH miniature swine are bred in a pathogen-free facility with defined MHC while maintaining minor antigen variation. Donors and recipients were matched for class II SLA and mismatched for class I SLA. Donors were positive for the expression of pig allelic antigen (PAA) to permit analysis of chimerism when transplanted into PAA negative recipients.

### Mixed Chimerism Protocol

**Figure 1:**
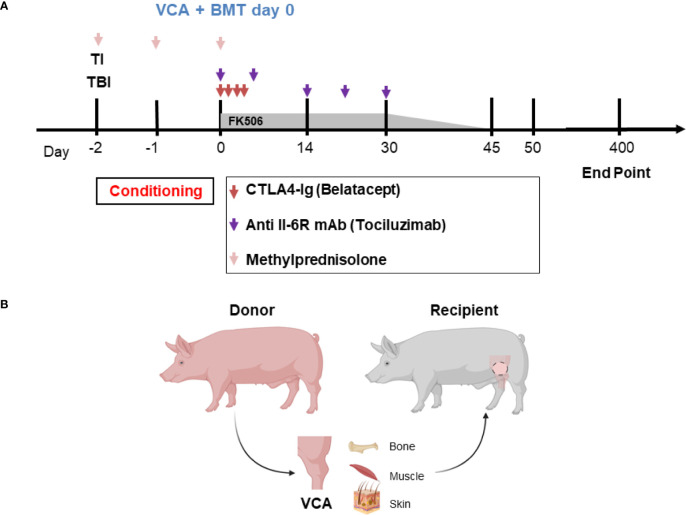
**(A)** Mixed chimerism protocol for immune tolerance. Recipient conditioning begins prior to transplantation with total body irradiation (TBI, day -2) and thymic irradiation (TI, day -1). Induction is performed with methylprednisolone (days -2, -1, 0) and FK506. Following vascularized composite allotransplantation (VCA), the donor is exsanguinated, and bone marrow is harvested for transplantation (BMT) under the cover of CTLA4-Ig (Belatacept^®^) and anti-IL6R mAb (Tociluzimab^®^). FK506 is then continued for 30 days (target trough levels: 10-15 ng/mL) before gradual tapering to discontinuation on day 45; further CTLA4-Ig (Belatacept^®^) and anti-IL6R mAb (Tociluzimab^®^) is given I.V. 20 mg/kg (on POD 2, 4, 6) and I.V. 10 mg/kg (on POD 7, 14, 21, 28) respectively. **(B)** Schematic of donor hindlimb VCA tissue components and placement in the recipient. Image created with BioRender.

The timeline and treatment protocol are depicted in **Figure 1A** and **Table 1**. Swine were conditioned with 300 cGy total body irradiation (TBI) and 700 cGy thymic irradiation (TI) both on day minus 2. Following donor bone marrow transplantation (target 1x10^9^ cells/kg) on the day of VCA surgery, the animals received co-stimulatory blockade using CTLA4-Ig (Belatacept^®^, Bristol-Myers-Squibb, Princeton, NJ) (20 mg/kg IV on days 0, 2, 4 and 6) and maintenance on intravenous FK506 for 30 days (target level 10-15 ng/mL). After day 30 the FK506 was reduced in a tapered dose to permit discontinuation by day 45. With the development of idiopathic pulmonary syndrome (IPS) in recipients 1 (R1) and R2, additional methylprednisolone (IM 10 mg) on days -2, -1, 0) and anti-IL6R mAb (Tociluzimab^®^, Genentech, San Francisco, CA.) (IV 10 mg/kg on days 0, 7, 14, 21, 28) were added to the remaining three recipients. Two untreated control swine (R6 and R7) received the same VCA across class-I-mismatch barriers without conditioning, bone marrow transplantation, or maintenance immunosuppression.

**Table 1:** Description of transplant characteristics.

Animal	Sex	Weight(kg, POD 0)	TBI (cGy)	TI (cGy)	Steroid	Costim Blockade (CTLA-4 Ig)	Anti-IL6R	Calcineurin Inhibitor	BM dose(cells x10^9^/kg)	Immunosuppression withdrawal (POD)	Multilineage Chimerism	Study Outcomes
R1	M	23.8	300	700	None	Yes	None	Tacrolimus	1.0	Not done, see outcomes	–	Early study removal, IPS, respiratory distress
R2	M	25.0	300	700	None	Yes	None	Tacrolimus	1.0	Not done, see outcomes	–	Early study removal, IPS, respiratory distress
R3	M	21.9	300	700	Yes	Yes	Tocilizumab	Tacrolimus	1.0	45	Yes	Immunosuppression free VCA tolerance
R4	M	10.7	300	700	Yes	Yes	Tocilizumab	Tacrolimus	1.0	45	Yes	Immunosuppression free VCA tolerance
R5	M	19.0	300	700	Yes	Yes	Tocilizumab	Tacrolimus	1.0	45	Yes	Immunosuppression free VCA tolerance
R6	M	38.0	None	None	None	None	None	None	None	–	–	VCA rejection
R7	M	38.0	None	None	None	None	None	None	None	–	–	VCA rejection

Total body irradiation (TBI), thymic irradiation (TI), centi-gray (cGy), bone marrow (BM), idiopathic pulmonary syndrome (IPS).

### VCA Transplantation

Osteomyocutaneous VCAs consisting of the distal femur, proximal tibia, muscle cuff, and an island of vascularized skin were performed as previously described and shown in **Figure 1B** (12). Briefly, the donor flap consisting of the distal femur, knee joint, proximal tibia, fibula, thigh muscles and skin paddle was harvested on a femoral vascular pedicle, which provided sufficient length and caliber for reliable microvascular anastomosis. A subcutaneous pocket was prepared in the abdomen of the recipient for allograft inset and reperfusion following microvascular anastomosis. End-to-end femoral vessel anastomosis was performed after flap inset. The flap was positioned with the skin paddle facing the dorsolateral abdominal wall where it is sutured to the recipient skin. The exteriorized skin component allowed for visual and histopathological monitoring of rejection. Donor bone marrow was manually collected from the spine and the long bones of the carcass, filtered, and prepared for intravenous infusion into the recipient at the end of the day of surgery.

Postoperatively, VCAs were monitored daily for clinical signs of rejection, defined by increased redness, swelling and epidermolysis. Tolerance was defined as rejection-free VCA survival for >100 days without immunosuppression, and with corresponding *in vitro* evidence of donor-specific unresponsiveness. VCA and host skin biopsies were also taken for histopathological analysis when clinical rejection was suspected, and at postoperative day (POD) 14, 30, 50, and every 50 days thereafter up to the experimental endpoint of POD 250, and secondarily extended to 400 days. Pathologic evaluation of VCA biopsies was performed on sections stained with hematoxylin and eosin (H&E) in accordance with the 2007 Banff Working Classification (13). The specificity and durability of immune tolerance was tested by applying split-thickness skin grafts at approximately day 150 on the dorsal region of recipients and measuring survival or rejection times. The grafted skin included autologous control, skin from the original VCA donor, and skin from a fully mismatched third-party swine. Split thickness skin grafts of 0.3mm thickness were harvested with a dermatome (Zimmer Biomet, Indiana, USA) and stored at -80°C in cryoprotective freezing media (Lonza, Basel, Switzeralnd) supplemented with 50% heat inactivated FBS (Gibco, Massachusetts, USA). At time of skin graft placement, donor, third party, and autologous skin grafts were thawed at room temperature and washed in normal saline before suturing in place.

### Chimerism Analysis

Peripheral blood mononuclear cells (PBMCs) were collected weekly to assess chimerism by flow cytometry. Monoclonal antibodies used include PAA (1038H-10-9, mouse IgM) and lineage markers to CD2 (MSA4, mouse IgG2a), CD3 (BB23-8E6, mouse IgG2a), CD4 (4-12-4, mouse IgG2b), CD8a (76-2-11, mouse IgG2a), CD21 (B-ly4, mouse IgG1), CD44 (IM7, rat IgG2b), CD79a (HM47, mouse IgG1), CD172a (74-22-15, mouse IgG1), and FOXP3 (FJK-16s, rat IgG2a). Gating strategy used for characterizing chimeric populations included PAA+ subsets of the following markers: T-lymphocytes (CD3+), B-lymphocytes (either CD3-, CD21+ or CD79a+), Myeloid Cells (CD172a+), T-regs (CD3+, CD4+, FOXP3+), Memory T-cells (CD3+, CD44hi, CD2hi). Flow cytometry data was collected using a FACSVerse (Becton Dickinson, California) and analyzed with FlowJo analysis software (FlowJo, LLC, Ashland, OR, USA).

### Mixed Lymphocyte Reaction

Mixed lymphocyte reactions (MLRs) were performed to test T cell allo-responsiveness of transplanted swine (14). Briefly, recipient PBMCs were labeled with carboxyfluorescein succinimidyl ester (CFSE) proliferation dye (BioLegend, San Diego, CA) for 10 minutes at 37°C, washed twice in complete RPMI, and plated at a concentration of 4x10^6^ cells/ml in 96-well flat-bottom tissue culture treated plates (Corning, NY). Stimulators cells consisting of either donor, or third-party swine antigen-presenting cells (APCs) were irradiated at 3000rads. Irradiated stimulators were co-incubated with CFSE labeled recipient cells at a 1:1 ratio for 96 hours at 37°C in 5% CO_2_. After incubation, cells were labeled with fixable viability dye eFluor 780 (eBioscience, ThermoFisher Scientific, Waltham, MA) followed by surface staining with anti-swine CD3 antibody. Samples were acquired with a FACSVerse (Becton Dickinson, California) and data were analyzed with FlowJo analysis software (FlowJo, LLC, Ashland, OR, USA). Viable CD3+ cell populations were assessed for CFSE fluorescence. Proliferation of recipient cells after co-incubation with stimulators was determined by dilution of the CFSE dye (**Figure 6A**).

### Cytokine Analysis to Assess Inflammatory Response

The capacity of peripheral blood cells to secrete cytokines was analyzed by intra-cytoplasmic staining. Recipient PBMCs were incubated for 5 hours with Cell Stimulation Cocktail (Thermo Fisher Scientific, Waltham, MA) and BrefeldinA (Thermo Fisher Scientific, Waltham, MA) solution, to block cytokine secretion, and washed in phosphate buffered saline. Thereafter, the cells were first incubated with surface marker antibodies such as PAA, CD3, CD4, and CD8, before fixation and permeabilization (Thermo Fisher Scientific, Waltham, MA) to allow entry of anti-IL-10 (clone 945A-1A9-26C2, mouse IgG1). The cells were acquired with a FACSVerse flow cytometer (Becton Dickinson, Franklin Lakes, NJ), and data were analyzed with FlowJo (FlowJo LLC, Ashland, OR).

### Donor Specific Antibody Detection

The presence of donor specific antibody in peripheral blood was tested using serum from recipient animals obtained at 50-day intervals. Recipient sera were heat-inactivated and incubated with donor, recipient and third-party PBMCs. The binding of serum IgG antibodies to B and T lymphocytes was then analyzed by flow cytometry using FITC-conjugated goat anti-swine IgG (heavy and light chain reactive; Seracare, Milford, MA). Samples were acquired with a FACSVerse flow cytometer (Becton Dickinson, Franklin Lakes, NJ), and data were analyzed with FlowJo (FlowJo LLC, Ashland, OR).

## Results

### Clinical and Histological Results of Long-term VCA Survival

**Figure 2:**
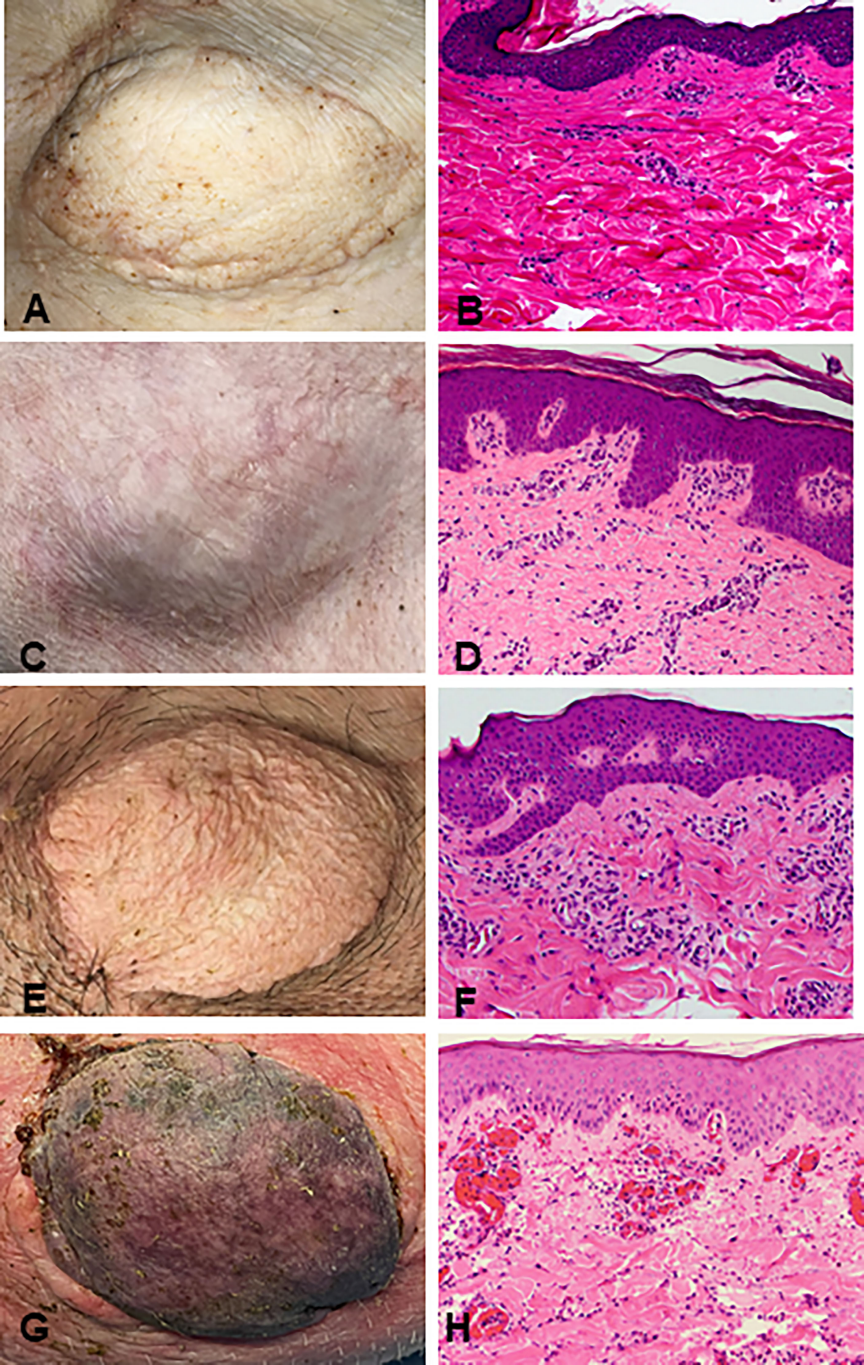
Clinical assessment of successful VCA tolerance. Images of tolerant VCAs from R3 **(A)**, R4 **(C)**, and R5 **(E)** taken on POD 251, 387, and 250, respectively, compared to a representative rejected VCA from untreated control R6 on POD 11 **(G)**. H&E staining of VCA biopsies from R3 **(B)**, R4 **(D)**, and R5 **(F)**, taken on POD 151, 387, and 250 respectively, demonstrating absence of rejection (Banff Grade 0). Whereas H&E staining of VCA skin from class I mismatch untreated control R6 shows focal epidermal necrosis and capillary thrombosis (Banff Grade IV) on POD 11 **(H)**. (POD, post-operative day; VCA, vascularized composite allograft).

Recipients R1 and R2 developed an idiopathic pneumonia-like syndrome (IPS) early post-transplantation and were euthanized on POD 36 and 39 respectively. These animals showed no signs of early rejection but were still receiving maintenance immunosuppression at end of study. Subsequent recipients were treated with methylprednisolone preoperatively and Tocilizumab^®^ postoperatively. As a result, recipients R3, R4, and R5 survived and had viable accepted grafts for up to 400 days (study end point) (**Figures 2A–F**). R4 developed a minor rejection episode on day 256 that resolved spontaneously. It is important to note that none of the recipients R3, R4 and R5 showed any signs of cutaneous or systemic GVHD at any time point post-transplantation. Therefore, three of five VCAs placed in swine (n=3) treated with our mixed chimerism protocol achieved long-term, immunosuppression-free survival. MHC class-I-mismatch VCA recipients that did not receive conditioning, bone marrow transplantation, or maintenance immunosuppression promptly rejected their transplants by day 11 (**Figures 2G, H**)

### Stable Chimerism in the Absence of Ongoing Immunosuppression

**Figure 3:**
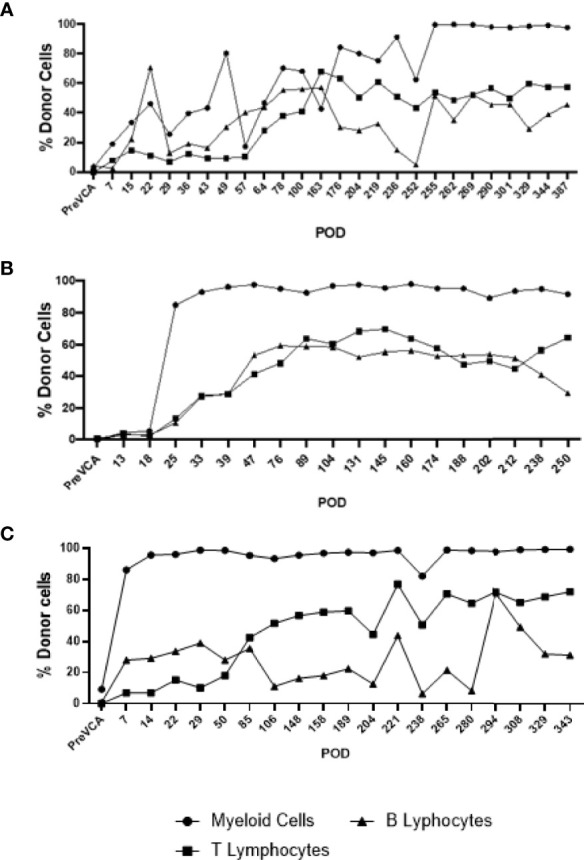
Long-term multi-lineage mixed chimerism in recipient animals R4 **(A)**, R3 **(B)** and R5 **(C)**. Levels of donor chimerism in myeloid cells were >80%, whereas B and T cell populations ranged between 30 to 60%.

**Figure 4:**
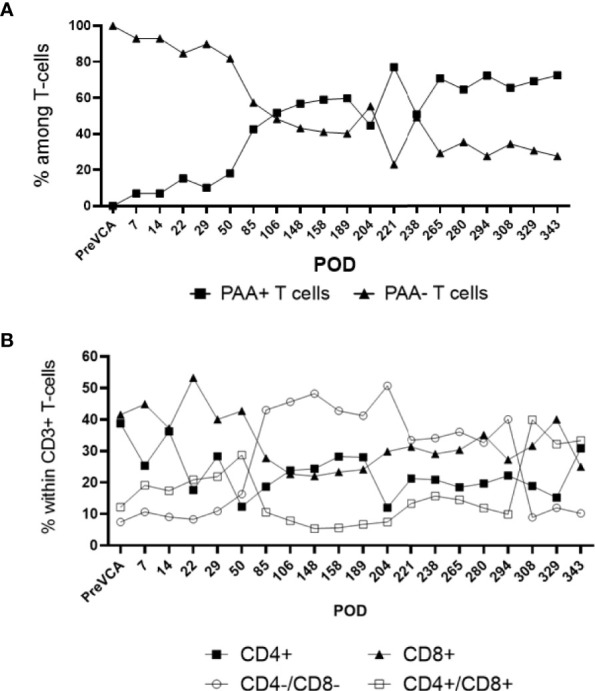
Sub-analysis of T cell subsets in multi-lineage mixed chimerism. Post-transplantation, as bone marrow reconstitution progresses, the percentage of donor (PAA+) and recipient (PAA-) T cells approaches equilibrium at around POD100, remained stable and varied between 50 to 70% of donor T cells to experimental endpoint **(A)**. The population of double negative (CD4-/CD8-) T cells increase from around POD 50 onwards (following cessation of FK506) and remained at about 20 to 40% of overall CD3+ T cell to experimental endpoint **(B)**. The results shown were obtained in one tolerant swine and are representative of all tolerant swine tested individually.

Recipient conditioning led to minimal leukocyte depletion in the peripheral blood and both myeloid and lymphoid cell compartments were fully restored within 28 days post-transplantation (**Supplementary Figure 1**). Importantly, all recipients displayed multilineage mixed hematopoietic chimerism during the entire duration of the study (400 days), as detected by staining with anti-PAA antibody (15), including all myeloid and lymphoid subsets (**Figure 3**). Virtually all recipient myeloid cells were replaced by donor cells (**Figure 3**). Donor chimerism ranging from 30-60% donor cells was detected for all other leukocyte subsets, including B and T lymphocytes (**Figure 3**). The frequencies of recipient (PAA^-^) T cells decreased progressively while the proportion of donor (PAA^+^) T cells increased until around day 100 (**Figure 4A**). Subsequently, the percentages of donor chimerism among T cells remained stable varying between 50-70% (**Figure 4B**).

### Tolerance of VCAs Was Donor-Specific

**Figure 5:**
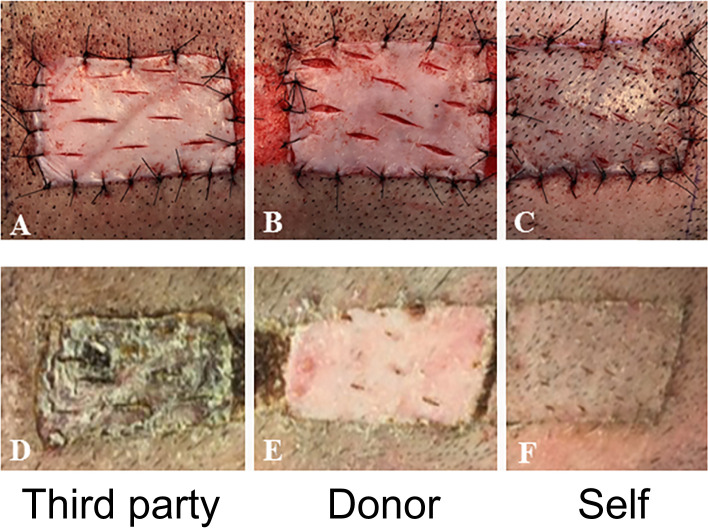
*In vivo* test of immune competence by placement of split-thickness skin grafts from a third-party animal **(A)**, original donor **(B)**, and self **(C)** at POD 150 onto recipient swine tolerant of VCA (representative). By POD 14, there was complete rejection of the skin graft from the third-party animal **(D)** whereas that from the donor **(E)** and self **(F)** had taken.

At day 150 post-transplantation, each long-term recipient received 3 split-thickness skin grafts: an autologous graft, one from the VCA donor, and one skin graft from a third-party swine (MHC-mismatched to both donor and recipient). Both autologous and donor-derived skin grafts were accepted without any immunosuppression until the end of the study. In contrast, all third-party skin grafts were rejected within 9 days after their placement (**Figure 5**). These results demonstrate that tolerance of VCAs was donor specific, yet the animals remained immunocompetent.

### Tolerance of VCAs Was Associated With Lack of Inflammatory T and B Cell Alloresponses

Next, we investigated the status and anti-donor responses of T and B cells in transplanted swine. No significant increase in the frequencies of memory T cells was detected post-conditioning and after VCA transplantation (**Supplementary Figure 2**), which indicates a lack of homeostatic expansion after leukodepletion and suggests a lack of sensitization to donor antigens after allotransplantation. In addition, it is noteworthy that we did not observe any expansion of FOXP3^+^ regulatory T cells (data not shown).

**Figure 6:**
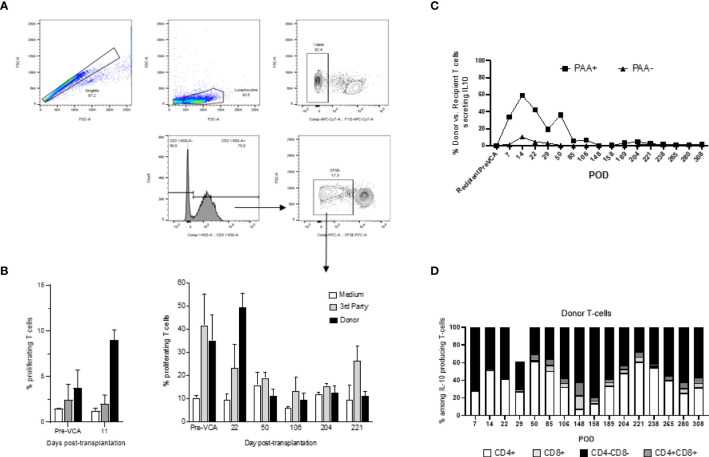
**(A)** Gating strategy for mixed lymphocyte reaction (MLR) assay. Cells analyzed in this assay were first gated as singlet events, followed by gating on the major lymphocyte population. Fixable viability dye negative events (viable) were analyzed for CD3+ expression. This population was characterized based on CFSE fluorescence, where proliferating T-cells were denoted as CFSE dim and plotted as a proportion of total CD3+ events. **(B)** Mixed lymphocyte reaction assay testing proliferation of T cells *in vitro* (left panel untreated controls, right panel tolerant chimeric recipients). Untreated control animals demonstrate increased T-cell proliferation against donor targets at time of rejection (left panel), whereas tolerant animals have decreased T-cell responsiveness towards the donor overtime (right panel). From POD 50 onwards, which corresponds more or less to the withdrawal of all immunosuppression and establishment of multi-lineage mixed chimerism, cellular proliferation against donor and self-antigens were comparable to medium, suggesting a state of immune unresponsiveness in peripheral blood (right panel). **(C)** Similar to **Figure 3** which showed equilibration of donor (PAA+) and recipient (PAA-) contribution to overall T cell populations, the contribution of IL-10 secreting T cells followed a similar pattern, approaching equilibrium at POD 85. Further analysis revealed that the majority of such IL-10 producing T cells are CD4-CD8- in nature **(D)**. The results shown in panel **(C, D)** were obtained in one tolerant swine and are representative of all tolerant swine tested individually.

Tolerance of VCAs was associated with a sustained lack of expansion/activation of donor-reactive T cells recognizing donor antigens through the direct allorecognition pathway (**Figure 6B**). Also, no donor-specific antibodies were detected at any given time point in serum samples of tolerant swine (data not shown). Finally, starting early post-bone marrow transplantation (day 7), we observed a substantial expansion of T cells of donor origin producing IL-10 cytokine (**Figure 6C**). Most of these IL-10 producing T cells were double negative (DN) CD4^-^CD8^-^ T cells (**Figure 6D**).

## Discussion

Vascularized composite allotransplantation has revolutionized reconstructive surgery with unparalleled aesthetic and functional results achieved in over 120 hand transplants and more than 40 face transplants over the last two decades. These innovative procedures represent a paradigm shift in our ability to reconstruct severe craniofacial deformities and restore function to amputees. Recently this approach has been extended to genitourinary tissue, with 4 successful penis transplants performed over the past three years (16). The VCA field is evolving past the proof-of-concept stage and into the need for reduction of the risk-benefit ratio to allow more widespread application of these revolutionary procedures. VCA patients are as susceptible as solid organ transplant recipients to complications from maintenance immunosuppression that include neoplasm, infection, and renal failure. As the population of long-term VCA recipients increases, so does the observed incidence of chronic rejection—an even more severe complication for which there is currently no solution other than re-transplantation (17).

In this context, the need for strategies to induce a state of immunologic tolerance in VCA is more important than ever. The mitigation of both acute and chronic rejection has been achieved with mixed chimerism regimens in large animals and in clinical renal transplant tolerance induction (6). Mixed chimerism is the coexistence of both donor and recipient hematopoietic cells in a homeostatic state that renders the recipient tolerant to the donor organs or tissues. Transplanted organs in individuals that have undergone a mixed chimerism based tolerance induction protocol appear pristine by biopsy even decades after transplantation.

In the current study, we have achieved an important milestone towards the goal of a tolerance protocol for VCA based on the induction of mixed hematopoietic chimerism. Such a protocol would need to be safe and reproducible for *fully mismatched* donor-recipient pairs, which are the most likely matching scenarios in clinical VCA transplantation where more closely matched pairs (such as with living related kidney donors) are not possible (i.e. relatives cannot donate hands or faces). A mixed chimerism protocol would also require a clinically relevant source of hematopoietic stem cells rather than cytokine-mobilized peripheral blood stem cells (CM-PBMC). CM-PBMC would be difficult to acquire in clinical VCA transplantation from a multi-organ donor, as the cytokines given to the donor may affect the suitability of the other organs for transplantation. Furthermore, it is impractical to delay the procurement teams for the various organs while performing leukopheresis to acquire the donor’s peripheral blood stem cells.

The use of CM-PBMCs as a stem cell source at supraphysiologic doses led to successful and stable mixed chimerism induction in the lab in our MGH VCA swine model, in which defined SLA permit mismatched pairs that mimic clinical mismatch situations (11). We obtained immunosuppression-free VCA tolerance across haploidentical mismatches (9) (analogous to a mother-daughter living related donor-recipient transplant pair). However, we encountered several problems when applying this regimen to fully mismatched swine pairs: on one hand, the full mismatch barrier required more ablative conditioning (for example higher doses of irradiation) to obtain stem cell engraftment in the recipient with large doses of CM-PBMC- resulting in early toxicity or graft versus host disease. Additionally, the number of bone marrow-derived stem cell and/or quality were not sufficient to engraft to produce stable chimerism, and the VCAs rejected as soon as chimerism disappeared (18).

The mechanisms underlying the induction and maintenance of mixed chimerism for immune tolerance to VCAs remain elusive. Our laboratory recently showed that recipients with VCAs performed directly after (day 3) establishment of mixed chimerism (from days 0 to 2) could be rendered tolerant (9). Neither T cell anergy nor peripheral regulation by regulatory T cells could be demonstrated to have a contributory role however, which suggests that local mechanism(s) might be responsible (9).

The features of the MGH swine herd allowed us to attempt to tease out the SLA responsible for the failure in fully-mismatched pairs and test the previously successful haploidentical protocol (using CM-PBMCs in both MHC class I-matched/class II-mismatched barriers and vice versa i.e. (MHC class I-mismatched/class II-matched barriers) (10). In these studies, the class II mismatched results mirrored the haploidentical transplant results- the CM-PBMC stem cell source resulted in stable, mixed chimerism and tolerance. Surprisingly, despite the CM-PBMC stem cell source again resulting in stable mixed chimerism, the MHC class I-mismatched VCAs were either partially or fully rejected. Some animals rejected only the allograft epidermis and healed the epidermis with host skin while remaining tolerant to the underlying dermis, whereas other animals fully rejected the entire VCA.

To solve the class-I-mismatch rejection problem, the following changes were made to the conditioning regimen in this study: irradiation of the whole body and thymus were increased, cyclosporine was replaced with FK506, co-stimulatory blockade with CTLA4-Ig was added, and anti-IL6R mAb was added, and a vascularized bone marrow component was included by incorporating the swine’s femur in the VCA. With this regimen, we achieved reproducible, stable mixed chimerism for the first-time using swine donor bone marrow as our stem cell source rather than CM-PBMCs. The stable mixed chimerism resulted in long-term immunosuppression-free tolerance of all components—epidermis included—of the VCAs.

It should be emphasized that the protocol tested here was not intended for clinical readiness. However, all conditioning medications (FK506, CTLA4-Ig, steroids, and anti-IL6R mAb) employed in this study are FDA-approved for clinical use in various other conditions, and these attributes (use of donor bone marrow rather than CM-PBMCs, as well as FDA-approved drugs), taken together, will be valuable for eventual clinical tolerance protocols across full deceased donor-recipient mismatches (mismatched at class I and class II). Furthermore, as machine perfusion and composite tissue preservation technologies advance towards clinical use, VCA recipients may benefit from a window of 72 hours for preconditioning, fitting within the time constraints of our protocol described here. The current clinical standard in tissue preservation, static hypothermic preservation (HP) in ice-cold preservation solution (4°C), cannot maintain tissue viability for more than a few hours, creating a technological bottleneck for VCA tolerance induction strategies. Berendsen et al. recently developed a novel sub-zero non-freezing protocol which preserves rat livers for 3 days with 100% post-transplant survival: three times the maximum possible with current clinical standard, HP (19) Adapting such a technology for use with composite tissues could help to overcome preconditioning timing in VCA.

In the current study, no significant increase in the number of memory T cells were detected post-conditioning and transplantation, which suggests a lack of homeostatic expansion and sensitization to donor antigens. We also did not observe any expansion of FOXP3^+^ regulatory T cells. MLR assays also showed a lack of expansion of activated donor specific T cells in tolerant swine. At the same time, the observation that some T cells were still activated *via in vitro* stimulation with donor APCs suggests that donor-reactive recipient T cells had not been deleted in tolerant swine. Interestingly, cytokine analysis revealed an early and sustained increase in the frequency of CD4^-^CD8^-^ double negative (DN) donor T cells producing IL-10, a regulatory cytokine. Similar DN T cells studied in rodents were previously shown to inhibit immune responses mediated by effector CD4^+^ and CD8^+^ T cells and prevented allograft rejection (20, 21) and GVHD (22, 23). In these studies, DN cells mediated their suppressive functions through down regulation of co-stimulatory molecules (CD80, CD86) by dendritic cells (DCs) and subsequent inhibition of their APC functions and by inducing apoptosis of DCs. In humans, DN T cells were shown to acquire MHC peptide complexes from DCs *via* trogocytosis and induce apoptosis of corresponding CD8^+^ cytotoxic T cells (24, 25). Together with our results, it suggests that DN T cells may contribute to VCA tolerance induced *via* mixed chimerism in our study.

While the regimen used in this study has not yet been tested in class II or haplo-mismatched pairs, the results demonstrate significant progress in class I tolerance compared to historical control animals treated with our previous conditioning regimen. Further work in the MHC class I disparate model will build on these encouraging preliminary results to examine which added variables (CTLA4-Ig, FK506, vascularized bone compartment) permitted both engraftment of bone marrow-derived stem cells and stable mixed chimerism, resulting in tolerance of the VCA.

## Data Availability Statement

The original contributions presented in the study are included in the article/**Supplementary Material**. Further inquiries can be directed to the corresponding author.

## Ethics Statement

The animal study was reviewed and approved by MGH Institutional Animal Care and Use Committee (IACUC).

## Author Contributions

AL, AA, GS, and ZN contributed to the design of experiments, animal surgeries and post-operative care, analysis of data, and writing of manuscript. AA, GS, IS, MG, and A-RG conducted *in vitro* tests, analyzed results, and participated in post-operative care of animals. MR contributed to the design of experiments, animal surgeries and post-operative care. IR and RC contributed to pathological analysis of histological samples and writing of manuscript. LL contributed to writing of manuscript. GB contributed to the design of *in vitro* experiments, analysis of data, and writing of manuscript. CC contributed to the design of experiments, animal surgeries and post-operative care, analysis of data, and critical revision of manuscript. All authors contributed to the article and approved the submitted version.

## Funding

This work was supported by the Sundry Fund of the Division of Plastic and Reconstructive Surgery, Massachusetts General Hospital, Shriners Hospitals for Children Boston, Musculoskeletal Transplant Foundation, and the Office of the Assistant Secretary of Defense for Health Affairs and the Defense Health Agency, Research, Development and Acquisition Directorate through the Reconstructive Transplant Research Consortium under Award No. (W81XWH-13-2-0060, W81XWH- 16-1-0702 and W81XWH-17-1-0680). Opinions, interpretations, conclusions and recommendations are those of the authors and are not necessarily endorsed by the DoD.

## Conflict of Interest

The authors declare that the research was conducted in the absence of any commercial or financial relationships that could be construed as a potential conflict of interest.

## Publisher’s Note

All claims expressed in this article are solely those of the authors and do not necessarily represent those of their affiliated organizations, or those of the publisher, the editors and the reviewers. Any product that may be evaluated in this article, or claim that may be made by its manufacturer, is not guaranteed or endorsed by the publisher.
