# Supplementary figures and images for: Tolerance of a Vascularized Composite Allograft Achieved in MHC Class-I-mismatch Swine via Mixed Chimerism
Source: Front Immunol. 2022 May 10;13:829406. doi: 10.3389/fimmu.2022.829406 (PMC9128064; doi:10.3389/fimmu.2022.829406)

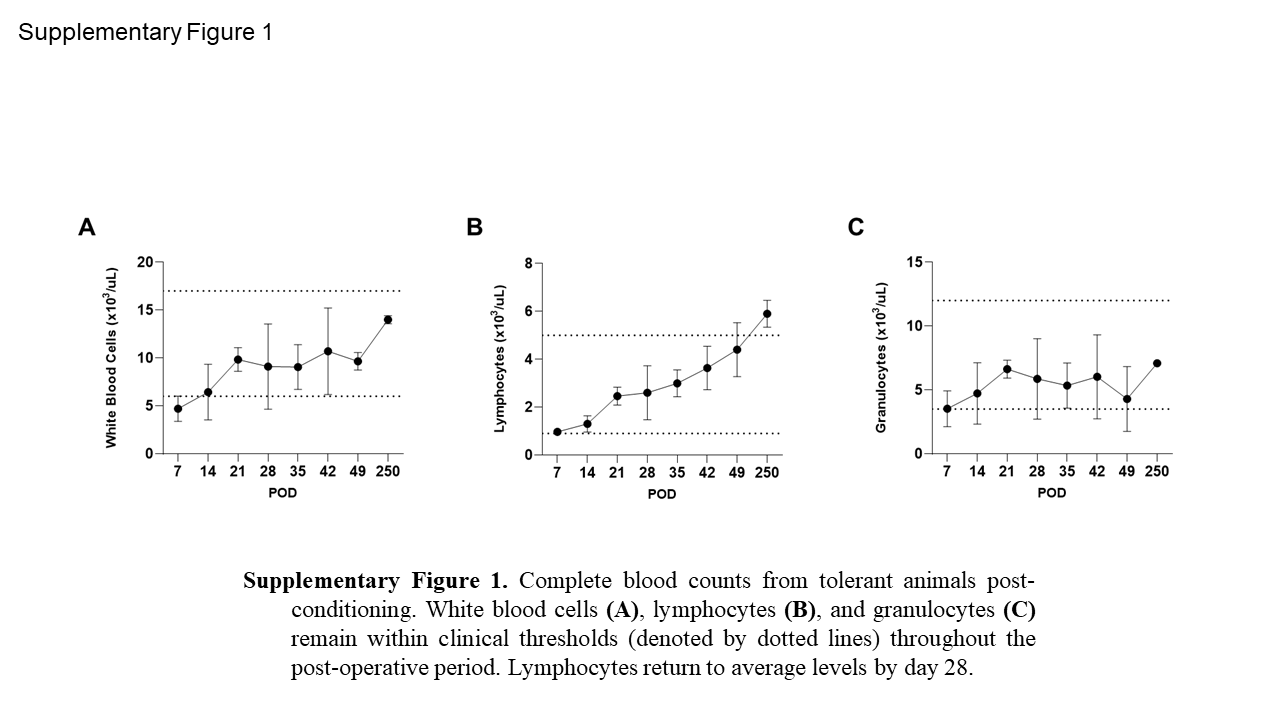

Supplement: Supplementary file 1 [file Image_1.tif]

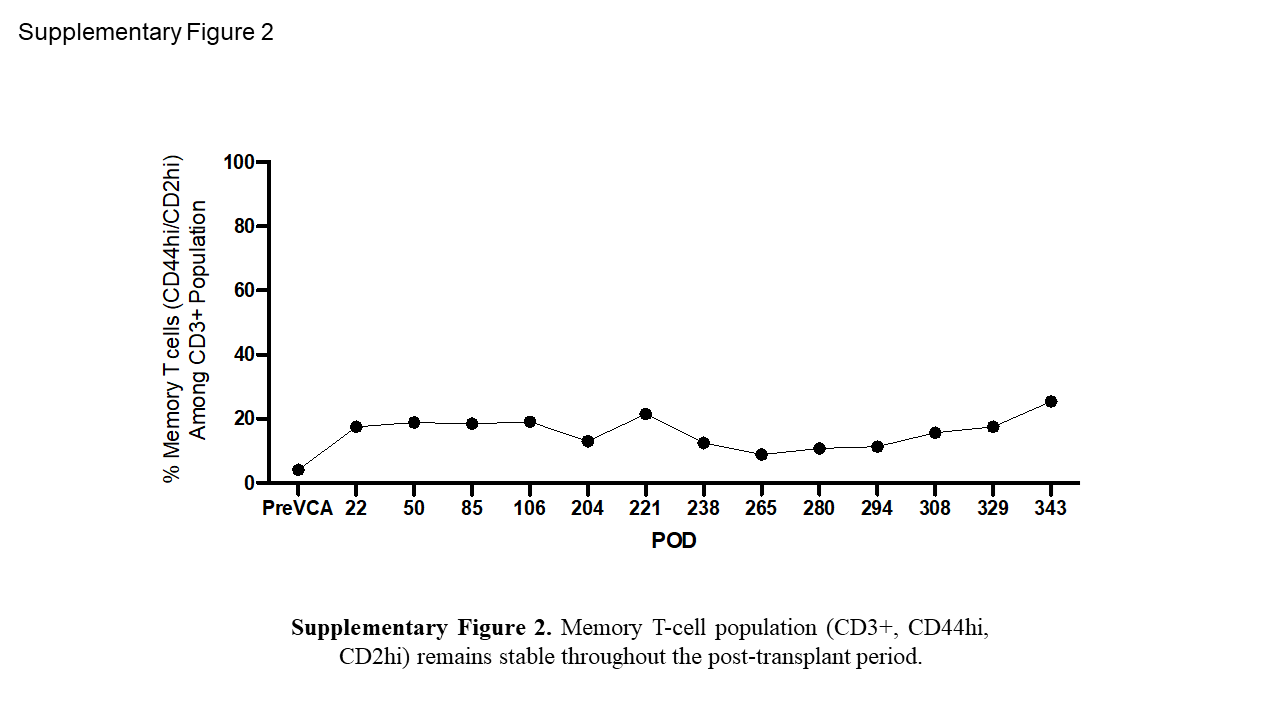

Supplement: Supplementary file 2 [file Image_2.tif]
